# Supplementary material for: A conserved and regulated mechanism drives endosomal Rab transition
Source: eLife. 2020 May 11;9:e56090. doi: 10.7554/eLife.56090 (PMC7239660; doi:10.7554/eLife.56090)
Supplement: Supplementary file 1. [file elife-56090-supp1.docx]

**Supplemental File 1a**

| **Strains** | **Genotype** | **Reference** |
| --- | --- | --- |
| BY4741 | MATa *his3∆1 leu2∆0 met15∆0 ura3∆0* | Euroscarf Library |
| BY4741 | MATa *his*3∆ *leu*2∆ *met*15∆ *ura*3∆ *mon*1∆::*kanMX*4 | Euroscarf Library |
| BY4741 | MATa *his*3∆ *leu*2∆ *met*15∆ *ura*3∆ *vps*30∆::*kanMX*4 | Euroscarf Library |
| CUY6517 | MATa *his*3∆ *leu*2∆ *met*15∆ *ura*3∆ *vps*21∆::*kanMX*4 *ypt*52∆::*natNT*2 | Cabrera et al., 2013 |
| CUY12510 | MATa *his*3∆ *leu*2∆ *met*15∆ *ura*3∆ *vps*21∆::*kanMX*4 *ypt*52∆::*natNT*2 *vps*30∆::*hphNT*1 | This study |
| CUY11940 | MATalpha *leu*2-3,112 *ura*3-52 *his*3-∆200 *trp*-∆901 *lys*2-801 *suc*2-∆9 *GAL ypt7*∆::*NatNT*2 *URA*3::pRS406*-YPT7pr-*mNeon*-(GGSG)*x3*-YPT*7*-YPT*7*term* | This study |
| CUY12183 | MATa *his*3∆ *leu*2∆ *met*15∆ *ura*3∆ *vps*21∆::*kanMX*4 *YPT*7::pRS406*-YPT7pr-*mNeon*-(GGSG)*x3*-YPT*7*-YPT*7*term* | This study |
| CUY12184 | MATa *his*3∆ *leu*2∆ *met*15∆ *ura*3∆ *ypt*10∆::*kanMX*4 *YPT*7::pRS406*-YPT7pr-*mNeon*-(GGSG)*x3*-YPT*7*-YPT*7*term* | This study |
| CUY12188 | MATa *his*3∆ *leu*2∆ *met*15∆ *ura*3∆ *ypt*53∆::*kanMX*4 *YPT*7::pRS406*-YPT7pr-*mNeon*-(GGSG)*x3*-YPT*7*-YPT*7*term* | This study |
| CUY12189 | MATa *his*3∆ *leu*2∆ *met*15∆ *ura*3∆ *ypt*52∆::*kanMX*4 *YPT*7::pRS406*-YPT7pr-*mNeon*-(GGSG)*x3*-YPT*7*-YPT*7*term* | This study |
| CUY12190 | MATa *his*3∆ *leu*2∆ *met*15∆ *ura*3∆ *vps*21∆::*kanMX*4 *ypt*52∆::*natNT*2 *YPT*7::pRS406*-YPT7pr-*mNeon*-(GGSG)*x3*-YPT*7*-YPT*7*term* | This study |
| CUY12191 | MATa *his*3∆ *leu*2∆ *met*15∆ *ura*3∆ *ypt*53∆::*kanMX*4 *ypt*52∆::*hphNT*1 *YPT*7::pRS406*-YPT7pr-*mNeon*-(GGSG)*x3*-YPT*7*-YPT*7*term* | This study |
| CUY11418 | MATalpha *his*3∆1 *leu*2∆0 *met*15∆0 *ura*3∆0 *lys*+ *can*1∆::*GAL1pr-Sce*I::*STE2pr-SpHIS*5 *lyp*1∆::*STE3pr-LEU*2 *YPT10pr*-sfGFP-*YPT10* *CCZ*1::3xmCherry-*hphNT*1 | This study |
| CUY2470 | BY4732; *CCZ1::TRP1-GAL1pr MON1::HIS3MX6-GAL1pr CCZ1::TAP-URA3* | Nordmann  et al., 2010 |
| CUY2675 | BY4732xBY4727 *VPS41*::*TRP*1-*GAL1pr* *VPS*41::*TAP-URA*3 *VPS39::KanMX*6-*GAL1pr* *VPS33*::*HIS3-GAL1pr VPS11*::*HIS3-GAL1pr VPS*16::*natNT*2-*GAL1pr VPS*18::*KanMX*6-*GAL1pr-3HA* | Ostrowicz et al., 2010 |
| CUY12521 | MATalpha *leu2-3,112 ura3-52 his3-∆200 trp-∆901 lys2-801 suc2-∆9* *GAL vps21::trp* | This study |
| CUY12522 | MATalpha *leu2-3,112 ura3-52 his3-∆200 trp-∆901 lys2-801 suc2-∆9 GAL ypt52::hphNT1* | This study |
| CUY12523 | MATalpha *leu2-3,112 ura3-52 his3-∆200 trp-∆901 lys2-801 suc2-∆9 GAL ypt53::hphNT1* | This study |
| CUY12524 | MATalpha *leu2-3,112 ura3-52 his3-∆200 trp-∆901 lys2-801 suc2-∆9 GAL ypt10::hphNT1* | This study |
| CUY12545 | MATalpha *leu2-3,112 ura3-52 his3-∆200 trp-∆901 lys2-801 suc2-∆9 GAL ypt52::hphNT1 ypt53::kanMX* | This study |
| CUY12546 | MATalpha *leu2-3,112 ura3-52 his3-∆200 trp-∆901 lys2-801 suc2-∆9 GAL ypt52::hphNT1 ypt10::kanMX* | This study |
| CUY12547 | MATalpha *leu2-3,112 ura3-52 his3-∆200 trp-∆901 lys2-801 suc2-∆9 GAL ypt52::hphNT1 vps21::kanMX* | This study |
| CUY12548 | MATalpha *leu2-3,112 ura3-52 his3-∆200 trp-∆901 lys2-801 suc2-∆9 GAL ypt53::hphNT1 ypt10::kanMX* | This study |
| CUY12549 | MATalpha *leu2-3,112 ura3-52 his3-∆200 trp-∆901 lys2-801 suc2-∆9 GAL ypt53::hphNT1 vps21::kanMX* | This study |
| CUY12550 | MATalpha *leu2-3,112 ura3-52 his3-∆200 trp-∆901 lys2-801 suc2-∆9 GAL ypt10::hphNT1 vps21::kanMX* | This study |
| CUY12618 | MATalpha *leu2-3,112 ura3-52 his3-∆200 trp-∆901 lys2-801 suc2-∆9 GAL ypt52::hphNT1 ypt53::kanMX ypt10::natNT2* | This study |
| CUY12619 | MATalpha *leu2-3,112 ura3-52 his3-∆200 trp-∆901 lys2-801 suc2-∆9 GAL ypt52::hphNT1 ypt53::kanMX vps21::natNT2* | This study |
| CUY12620 | MATalpha *leu2-3,112 ura3-52 his3-∆200 trp-∆901 lys2-801 suc2-∆9 GAL ypt10::hphNT1 vps21::kanMX ypt52::natNT2* | This study |
| CUY12621 | MATalpha *leu2-3,112 ura3-52 his3-∆200 trp-∆901 lys2-801 suc2-∆9 GAL ypt10::hphNT1 vps21::kanMX ypt53::natNT2* | This study |
| CUY12623 | MATalpha *leu2-3,112 ura3-52 his3-∆200 trp-∆901 lys2-801 suc2-∆9 GAL ypt52::hphNT1 ypt53::kanMX ypt10::natNT2 vps21::trp* | This study |
