# Supplementary material for: A conserved and regulated mechanism drives endosomal Rab transition
Source: eLife. 2020 May 11;9:e56090. doi: 10.7554/eLife.56090 (PMC7239660; doi:10.7554/eLife.56090)
Supplement: Supplementary file 2. [file elife-56090-supp2.docx]

**Supplemental File 2 – Plasmids used in this study**

| **Protein** | **Backbone** | **Reference** |
| --- | --- | --- |
| YPT7pr-mNeon-YPT7-YPT7term | pRS406 | This study |
| s.c. Mrs6 | pET30 | Gift from K.Alexandrov |
| s.c. Bet4-s.c. Bet2 | pCDF-DUET-1 | Thomas et al, 2016 |
| s.c. Gdi1 | pGEX-6P | Thomas et al., 2016 |
| Sec4 | pET24d-GST-TEV- | Lachmann et al., 2012 |
| Vps21 | pET24d-GST-TEV- | Lachmann et al., 2012 |
| Ypt1 | pET24d-GST-TEV- | Lachmann et al., 2012 |
| Ypt6 | pET24d-GST-TEV- | Lachmann et al., 2012 |
| Ypt7 | pET24d-GST-TEV- | Lachmann et al., 2012 |
| Ypt10 | pET24d-GST-TEV- | Lachmann et al., 2012 |
| Ypt31 | pET24d-GST-TEV- | Lachmann et al., 2012 |
| Ypt32 | pET24d-GST-TEV- | Lachmann et al., 2012 |
| Ypt52 | pET24d-GST-TEV- | Lachmann et al., 2012 |
| Ypt53 | pET24d-GST-TEV- | Lachmann et al., 2012 |
| d.m. GDI | pET28a His-SUMO- | This study |
| d.m. Rab5 | pET24d-GST-TEV- | This study |
| d.m. Rab7 | pET24d-GST-TEV- | This study |
| GST-pre-d.m. Mon1-d.m.Ccz1-3xFlag | pBIG1a- | This study |
| GST-pre-d.m. Mon1-d.m. Ccz1-3xFlag-d.m. CG8270 | pBIG1a- | This study |
| s.c. Yck3 | pHIS.Parallel1 | Hickey et a., 2009 |
