# Supplementary material for: A conserved and regulated mechanism drives endosomal Rab transition
Source: eLife. 2020 May 11;9:e56090. doi: 10.7554/eLife.56090 (PMC7239660; doi:10.7554/eLife.56090)
Supplement: Transparent reporting form [file elife-56090-transrepform.docx]

***eLife’s* transparent reporting form**

We encourage authors to provide detailed information *within their submission* to facilitate the interpretation and replication of experiments. Authors can upload supporting documentation to indicate the use of appropriate reporting guidelines for health-related research (see [EQUATOR Network](http://www.equator-network.org/%20)), life science research (see the [BioSharing Information Resource](https://biosharing.org/)), or the [ARRIVE guidelines](http://www.plosbiology.org/article/info:doi/10.1371/journal.pbio.1000412) for reporting work involving animal research. Where applicable, authors should refer to any relevant reporting standards documents in this form.

If you have any questions, please consult our Journal Policies and/or contact us: [editorial@elifesciences.org](mailto:editorial@elifesciences.org).

**Sample-size estimation**

- You should state whether an appropriate sample size was computed when the study was being designed
- You should state the statistical method of sample size computation and any required assumptions
- If no explicit power analysis was used, you should describe how you decided what sample (replicate) size (number) to use

Please outline where this information can be found within the submission (e.g., sections or figure legends), or explain why this information doesn’t apply to your submission:

For fluorescence microscopy in Figure 1 around 200 cells were analyzed for vacuole morphology per strain. Colocalization analyses were based on single planes. In general, fluorescently tagged proteins under control of the endogenous promotor have been used. Cells shown depict a representative example.

GST-Pull down assays in Figure 1: 9 out of the 11 Rab-GTPases from *Saccharomyces cerevisiae* have been tested for interaction with purified Mon1-Ccz1.

Mon-Ccz1 membrane association in Figure 2: Significance analysis were performed by two-tailed heteroscedastic t-test statistics.

All error bars shown in this paper depict the standard deviation of the performed experiments.

**Replicates**

- You should report how often each experiment was performed
- You should include a definition of biological versus technical replication
- The data obtained should be provided and sufficient information should be provided to indicate the number of independent biological and/or technical replicates
- If you encountered any outliers, you should describe how these were handled
- Criteria for exclusion/inclusion of data should be clearly stated
- High-throughput sequence data should be uploaded before submission, with a private link for reviewers provided (these are available from both GEO and ArrayExpress)

Please outline where this information can be found within the submission (e.g., sections or figure legends), or explain why this information doesn’t apply to your submission:

Analyses of fluorescence microscopy in Figure 1 relied on at least two biological replicates.

GST-Pull down assays in Figure 1 and 6: at least three biological repeats for every Rab-GTPase. Biological repeats in this case refers to independent purifications of GST-Rabs and Mon1-Ccz1.

Mon-Ccz1 membrane association in Figure 2 was performed with 3 biological repeats, membrane association in Figure S2 was performed with three technical repeats. Significance analysis were performed by two-tailed heteroscedastic t-test statistics.

Recruiter GEF-assays in Figure 3 and 4 with non-modified or phosphorylated Mon1-Ccz1 have been performed with at least two technical repeats per concentration of GEF and recruiter.

Proteoliposome Fusion Assay in Figure 5 has been performed with three technical repeats, membrane fraction was analysed from two of these assays (Fig 5D,E). These two assays were performed with different, independent preparations of Mon1-Ccz1.

**Statistical reporting**

- Statistical analysis methods should be described and justified
- Raw data should be presented in figures whenever informative to do so (typically when N per group is less than 10)
- For each experiment, you should identify the statistical tests used, exact values of N, definitions of center, methods of multiple test correction, and dispersion and precision measures (e.g., mean, median, SD, SEM, confidence intervals; and, for the major substantive results, a measure of effect size (e.g., Pearson's r, Cohen's d)
- Report exact p-values wherever possible alongside the summary statistics and 95% confidence intervals. These should be reported for all key questions and not only when the p-value is less than 0.05.

Please outline where this information can be found within the submission (e.g., sections or figure legends), or explain why this information doesn’t apply to your submission:

The mean and standard deviation were calculated for Figure 1B; Figure 2B, Figure 2 - Figure supplement 1; Figure 3D; Figure 4E, G; Figure 5C,E, Figure 5 supplement 1; Figure 6B

The information can be found in method part and source data.

(For large datasets, or papers with a very large number of statistical tests, you may upload a single table file with tests, Ns, etc., with reference to sections in the manuscript.)

**Group allocation**

- Indicate how samples were allocated into experimental groups (in the case of clinical studies, please specify allocation to treatment method); if randomization was used, please also state if restricted randomization was applied
- Indicate if masking was used during group allocation, data collection and/or data analysis

Please outline where this information can be found within the submission (e.g., sections or figure legends), or explain why this information doesn’t apply to your submission:

Does not apply.

**Additional data files (“source data”)**

- We encourage you to upload relevant additional data files, such as numerical data that are represented as a graph in a figure, or as a summary table
- Where provided, these should be in the most useful format, and they can be uploaded as “Source data” files linked to a main figure or table
- Include model definition files including the full list of parameters used
- Include code used for data analysis (e.g., R, MatLab)
- Avoid stating that data files are “available upon request”

Please indicate the figures or tables for which source data files have been provided:

Figure 1A; Figure 2A and Figure 2 Supplement 1; Figure 3B, D; Figure 4E, G; Figure 5C, 5E and Figure 5 supplement 1; Figure 6B.
